# Auditory evoked potential: a proposal for further evaluation in children with learning disabilities

**DOI:** 10.3389/fpsyg.2015.00788

**Published:** 2015-06-10

**Authors:** Ana C. F. Frizzo

**Affiliations:** Department of Speech Pathology, Paulista State University, Marília, Brazil

**Keywords:** auditory evoked potential, learning disabilities, P300 event-related potential, middle latency response, dyslexia

## Abstract

The information presented in this paper demonstrates the author’s experience in previews cross-sectional studies conducted in Brazil, in comparison with the current literature. Over the last 10 years, auditory evoked potential (AEP) has been used in children with learning disabilities. This method is critical to analyze the quality of the processing in time and indicates the specific neural demands and circuits of the sensorial and cognitive process in this clinical population. Some studies with children with dyslexia and learning disabilities were shown here to illustrate the use of AEP in this population.

## Introduction

In Brazil, approximately 60% of the children in fourth grades of elementary school do not have the necessary basic competences to learn to read and write, and approximately 20% of the children remain illiterate during this period (INEP, 2003). The causes of poor school performance are often diverse and may be overlapped in children with school difficulties. Problems related to poor education, culture, physical or mental health, genetic or environmental reasons are generally associated with difficulties in the acquisition of scholar skills.

This scenario justifies the interest of health and education professional efforts to understand reading and writing disorders. In the current context, support programs have been developed in order to foster of these children’s ability to read and write. A broad approach by a multidisciplinary team of professionals may reach tangible results to compensate these disorders and changes in the Brazilian educational panorama.

Scientific research has focused on the understanding of the processes involved in general learning (Bledowski et al., 2009; Möhler, 2009; Nippold and Sun, 2009; Op de Beeck and Baker, 2009; Polk et al., 2009; Yadon et al., 2009) and pathological brain processes involved in learning disorders. The major investments in the area are related to dyslexia highlighting electrophysiological studies (Chermak and Musiek, 1994; Arehole et al., 1995; Purdy et al., 2002; Horowitz-Kraus and Breznitz, 2008; Sebastian and Yasin, 2008; Andreadis et al., 2009; Hämäläinen et al., 2013).

Thus, auditory evoked potential (AEP) has shown to be useful diagnostic tools for the functional assessment of the auditory system (Alain et al., 2013). The essential clinical application is in understanding auditory attention cognitive skills, auditory discrimination and memory. The study of AEP amplitude and latency allows the measurement of neuroelectric activity at each point of the auditory pathway in the nervous system (Pratt, 2007).

Auditory middle latency responses (AMLR), for instance, have shown to be an appropriate method to assess superior neural structures of hearing and language. Researchers have shown that these potentials relate to the nuclei and the auditory pathways situated in the thalamo-cortical region and the primary auditory cortex (Hall, 2006). The recording of these potentials reflect the cortical activities involved in the primary (recognition, discrimination, and figure-ground) and non-primary (selective attention, auditory sequence, and audio-visual integration) listening skills (Pratt, 2007; McPherson et al., 2008).

Another AEP measurement, Long Latency Evoked Potentials or Cognitive Potentials-P300, is related to sensory and cognitive functions. It represents conscious recognition, attention and auditory discrimination of the acoustic characteristics of the stimuli (tones and speech). P300 is recorded consciously when a deviant and random stimulus is detected among a series of standard stimuli by the subject in evaluation—oddball paradigm (Hall, 2006; McPherson et al., 2008).

The learning of spoken and written language implies the incorporation of acoustic elements and the representation of their phonetic-phonological characteristics of a language. These potentials improve a precise observation of auditory and speech processing (Pratt, 2007). In general, they are easy-to-apply tests, and poorly explored by health and education professionals. Thus, this paper highlights the application of AEP in school children with reading and writing disorders.

## Perspective Study of AEP

The information presented in this paper demonstrates the author’s experience in previews cross-sectional studies conducted in Brazil, in comparison with the current literature. Over the last 10 years, AEP has been used in children with learning disabilities. This method is critical to analyze the quality of the processing in time and indicates the specific neural demands and circuits of the sensorial and cognitive process in this clinical population. Some studies with children with dyslexia and learning disabilities were shown here to illustrate the use of AEP in this population.

## Auditory Middle Latency Evoked Responses

Auditory middle latency responses are promising auditory tests that allow the identification of functional deficits of the central auditory pathways, and the cerebral hemispheres in school children with reading and writing learning disorders. The recording of these potentials ensure visualization of the electrical activity of the primary auditory cortex and the auditory thalamus-cortical pathways, from the observation of a sequence of waves, negative (N) and positive (P). Na, Pa, Nb, Pb occur in 10–80 ms intervals after stimuli (McPherson et al., 2008).

Auditory middle latency responses can also be used to investigate clinical conditions related to auditory processing disorder, contributing to neurodiagnosis and improving the understanding of Central Auditory Nervous System. Their main clinical advantages are accuracy and objectivity. They are not dependent on patient response and can be very useful in the evaluation of children (Pratt, 2007).

The Pa wave is the most robust component in AMLR. The Pa latency shows the processing time of the auditory information to the thalamo-cortical pathways and auditory cortex (Hall, 2006). The Na–Pa amplitude is also important information as AMLR shows the specific electrical activity accumulated in the auditory cortex.

**FIGURE 1:**
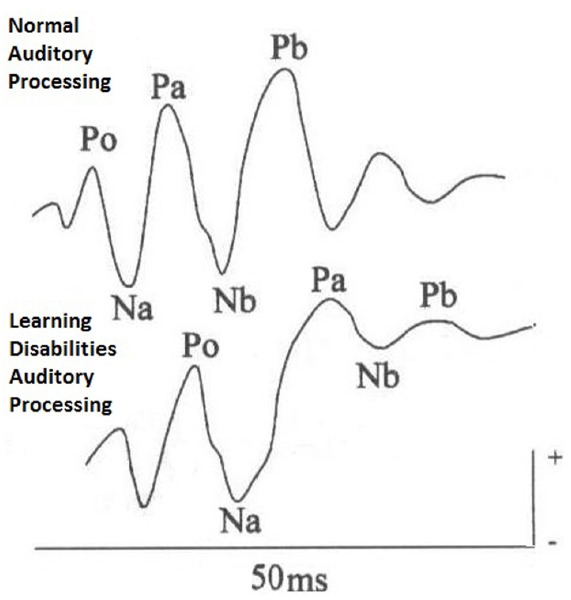
**Auditory middle latency responses (AMLR) traces—normal processing × non-normal auditory processing**.

The most significant differences observed between groups of children with and without reading and writing disorders relate to the components of Na and Nb, and Pa wave (Figure 1), but specifically to the delayed latency measures in contralateral pathway to the left (Frizzo et al., 2012). Children with learning disorders showed delayed latencies for Na wave in the left hemisphere (Arehole et al., 1995; Purdy et al., 2002). Furthermore, differences in morphology of the waves Pa, Nb and Pb were observed in children with school complaints (Frizzo, 2004; Table 1).

**TABLE 1:** Ipsi and contralateral via comparisons between groups: control × learning disabilities (*n* = 30)**.

**Variable**	**Group**	**Ear**	**Hemi**	**Group**	**Ear**	**Hemi**	**Estimated difference**	**UL CI 95%**	**LL CI 95%**	***P*-value**
**Lat_Pa**										
	C	R	R	S	L	R	–3.30	–6.55	–0.05	0.04*
	C	R	R	S	L	L	–3.36	–6.61	–0.10	0.04*
	C	R	L	S	L	R	–3.43	–6.68	–0.17	0.03*
**Lat_Nb**										
	C	R	R	S	L	L	–4.95	–9.02	–0.89	0.02*
	C	R	L	S	L	L	–4.20	–8.27	–0.13	0.04*
	C	L	R	S	R	L	–4.35	–8.42	–0.28	0.03*
	C	L	L	S	R	L	–4.69	–8.76	–0.63	0.02*
**Ampl Na-Pa**										
	C	L	R	S	L	L	0.15	0.00	0.30	0.04*

C, control group; S, study group; Lat, latency; Ampl, amplitude; R, right; L, left; Hemi, hemisphere; LL, lower limit; UL, upper limit; CI, confidence interval; RR/LL, ipsilateral way; RL/LR, contralateral way. *p ≤ 0.05—Kruskal–Wallis Test. **Frizzo (2004).

The essential hypothesis is that the dysfunction of the left contralateral auditory pathway can produce difficulties in sound decoding in children with learning disabilities. Consequently, this dysfunction also can lead to losses in the association of linguistic components with visual components. Or even, it can induce flaws in associations of auditory information with the linguistic information at primary and non-primary cortical areas.

The error in processing speed may explain, for instance, the inability to read and write. It would be consistent with the functional inefficiency of the left hemisphere and its integration of auditory and non-auditory information. The AMLR assessment evidenced these findings and supported this hypothesis. Currently, the use of AMLR in the literature is a new approach in Audiology area and represents new perspectives because the responses show information about neuroelectrical transmission along the auditory central pathways *in vivo*.

## Long Latency Auditory Evoked Potentials or Cognitive Potentials-P300

Long Latency Auditory Potential or Cognitive Potentials are bioelectric responses of the thalamus and cortex activity. These responses correspond to a series of peaks with negative (N) and positive (P) polarities generated along the auditory pathway, by one or more events. It is possible to analyze these components as for their latency and amplitude (Hall, 2006). The registration of these potentials shows a sequence of peaks with negative-positive-negative-positive polarity (N1-P2-N2-P3) in 80 and 350 ms intervals after stimuli (McPherson, 1996; Kraus and Mcgee, 2002).

The observation of the component complex of exogenous or sensory waves P1-N1-P2 is related to the perception of temporal and acoustic stimulus in the central auditory system, right from the onset of auditory cortical processing (Martin et al., 2007). The P300 wave is the greatest positive where increases in latency or decrease in amplitude is evidence of clinical and subclinical problems. If the P300 wave is small and delayed, there is evidence of a deficit in the cognitive processing (Hall, 2006).

P300, cognitive or endogenous potential is associated to mental function of perception and represents the physiological phenomena related to auditory attention, discrimination, integration and memory (Kraus and Mcgee, 2002). The assessed patient receives a task for conscious recognition of changes in auditory sensory stimuli. Then, the distinction between a stimulus presented standardly or a deviant stimulus presented randomly generates the P3 component or P300 (McPherson, 1996).

Electrophysiological studies have shown physiological deficits in children with learning disorders (Purdy et al., 2002; Regaçone et al., 2014) and dyslexia (Table 2; Lippanen and Lyytinen, 1997; Bonte and Blomert, 2004; Oliveira et al., 2013). Such deficits result in brain cognitive dysfunction related to selective attention, working memory or language processing. In general, we observed delayed values of the components in dyslexic children’s group compared with children without dyslexia.

**TABLE 2:** Statistical Analysis of Cognitive Potentials-P300 and comparison between groups: control × dyslexics**.

**Orelha/eletrodo**	Media (μV)		Control (*n* = 12)		Dyslexics (*n* = 12)		***P*-value**
			**Mean**	**SD**	**Mean**	**SD**	
RE Cz	Interamplitude	N2-P3	–6,86	9.72	6.36	3.08	0.0010*
LE Cz	Interamplitude	N2-P3	–6.75	12.53	6.16	0.77	0.0010*
RE Fz	Interamplitude	N2-P3	–5.12	11.02	6.43	2.18	0.0404*
LE Fz	Interamplitude	N2-P3	–3.79	11.73	5.60	4.65	0.0079*

RE, right ear; LE, left ear; SD, stander deviation; Fz, frontal midline; Cz, central midline. *p < 0.05—t-test. **Regaçone et al. (2014).

The latency was delayed especially regarding N1 and P2 (Lippanen and Lyytinen, 1997), N2 (Mazzotta and Gallai, 1992) and P3 or P300 components (Holcomb et al., 1986; Kujala and Naatanen, 2001; Maciejewska et al., 2012). Delayed latencies of N1 and P2 components may be associated with failures related to the auditory processing onset, but specifically to deficits in auditory cortical information synchronization associated to auditory attention factors (Lippanen and Lyytinen, 1997). The delayed N2 and reduced amplitude in students with dyslexia reflects difficulties in passive and automatic auditory sensory processing responsible for auditory perception, attention and discrimination of sounds (Ortiz et al., 1990; Näätänen, 1992; Ceponiene et al., 2002). The decrease in the amplitude of the P3 wave can be related to reduced amount of electrical activity involved in processing of secondary areas—complex auditory process related to auditory-linguistic processing (Ortiz et al., 1990). Other hypotheses that justify the changes observed in Cognitive Potentials-P300 in children with dyslexia have been raised, such as losses in phonological processing associated with impairments in specific cognitive areas for temporal coding and difficulties in processing the temporal fine structure of sounds (Sauer et al., 2006; Yeung and Werker, 2009; Reid et al., 2010).

**FIGURE 2:**
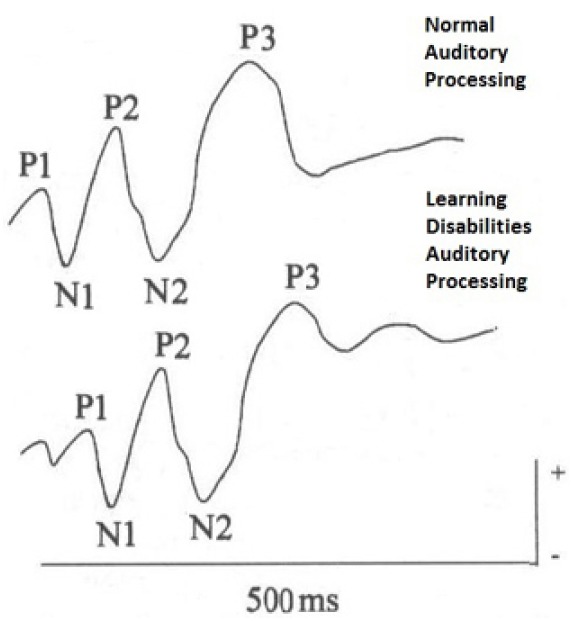
**Cognitive Potentials-P300 traces—normal processing × non-normal auditory processing**.

There are numerous studies with children with learning disabilities using LLAEP regarding the AMLR dyslexia. Most differences have been identified in latency components of the waves, but especially in the amplitude of late cognitive components (Ortiz et al., 1990; Lippanen and Lyytinen, 1997; Sauer et al., 2006; Hämäläinen et al., 2015; Figure 2). The reports infer disabilities in auditory attention, discrimination, storage and temporal processing and/or auditory information and their association with linguistic information, which can be a contributing factor to reading difficulties in dyslexic children.

## Final Considerations

The use of AEP measures does not aim the etiologic diagnosis of dyslexia or learning disabilities. However, it provides significant additional measure on the functioning of the auditory system and the temporal processing of linguistic information. Thus, they are an important assessment for the auditory development follow-up and the readiness for the reading and writing.

The electrophysiological findings of this study may suggest anatomical and/or functional flaws in students with learning disabilities. The evaluation of auditory disorders through AEP assessment complements the diagnosis of school children with learning disorders. This test may provide the opportunity for a thorough treatment planning, for an auditory-linguistic training and improvement of auditory skills, necessary for the acquisition of reading and writing.

The detection and early intervention in children with learning disabilities is essential to mitigate the negative impact on academic and social life in this population. However, more investment in research in this area is needed to search for more accurate information on the functioning of the auditory pathway in this population, and carry out further investigation of the auditory processing and linguistic stimuli in school children with learning disabilities.

### Conflict of Interest Statement

The author declares that the research was conducted in the absence of any commercial or financial relationships that could be construed as a potential conflict of interest.
